# Editorial: Alternative Polyadenylation in Development and Disease

**DOI:** 10.3389/fgene.2022.936960

**Published:** 2022-07-01

**Authors:** Athma A. Pai, Yang I. Li, Paolo Provero

**Affiliations:** ^1^ University of Massachusetts Medical School, Worcester, MA, United States; ^2^ Department of Human Genetics, The University of Chicago, Chicago, IL, United States; ^3^ Department of Neurosciences, University of Turin, Turin, Italy

**Keywords:** polyadenylation, 3' UTR, gene regulation, RNA-binding proteins, mRNA processing

Alternative polyadenylation, i.e., the production of mRNA isoforms with 3’ UTRs of different lengths or compositions depending on cell type, developmental stage, and the cell environment, has been shown to increase the astonishing complexity of gene regulation in eukaryotes. This Research Topic aimed to bring together contributions tackling the most pressing questions related to this phenomenon, namely the mechanisms behind this mode of regulation, its functional consequences, and the development of specific experimental and computational methods for its investigation.

The contribution by Wei and Lai is concerned with the molecular mechanisms of APA. Their review focuses on the role of the conserved family of ELAV/Hu RNA-binding proteins in regulating APA, in particular by influencing the characteristic lengthening of 3’ UTR isoforms observed in neural tissues. The review also highlights the close connection between APA and alternative splicing, pointing to a crucial role of RNA-binding proteins in multiple aspects of gene regulation.

The other contributions to the Topic focus on APA function from several complementary points of view. Experimental methods able to specifically investigate the functional consequences of APA are especially needed. In this Research Topic, Bae and Miura propose a CRISPR-based gene editing method for the deletion of long 3’ UTR isoforms in mouse embryonic stem cell-derived neurons, allowing a precise experimental assessment of the specific phenotypic consequences of APA. Their work also establishes embryonic stem cell-derived neurons as a convenient system for the study of post-transcriptional regulation in neural cells.

A topic closely related to the functional relevance of APA is its evolutionary conservation. While APA has been studied in several model organisms, its existence and relevance in less studied species has yet to be elucidated. Scharfen et al. demonstrate the high prevalence of APA in an alga, *Cyanidioschyzon merolae*, highly diverged from yeast and animals. Notwithstanding the strong selective pressure experienced by this species to simplify its RNA-processing machinery, leading to widespread loss of introns and reduced splicing, APA occurs in a proportion of transcripts similar to that in animals. This observation strengthens the case for a fundamental and deeply conserved role of this regulatory modality to diversify the transcriptome. Methodologically, this contribution highlights the advantages of using modern high-throughput long-read sequencing techniques for the investigation of transcript structure and specifically of APA.

An important role of APA is the regulation of mRNA stability and its subcellular localization. Guvenek *et al.* compared mRNA stability in different human cell lines. Specific transcripts, characterized by high GC content and more stable secondary structure, were shown to have higher stability in neuronal cells, and such cell type specificity extended to alternative isoforms generated by APA. This work suggests that the control of mRNA stability, including by APA, is a key factor in determining neuronal cell identity.

Arora *et al.* focused their review on one of the most elusive effects of APA, namely the differential subcellular localization of isoforms differing in the expressed 3’ UTR length and/or composition. This work underlined the need for an increased understanding of the “zip codes,” located in the UTRs, which determine the subcellular localization of mRNA to elucidate the causal mechanisms leading from APA to differential localization.

Together, the contributions in this Research Topic describe new discoveries about APA that are likely just scratching the surface of what remains to be learned. Despite increasing interest and appreciation of the roles that APA plays to shape transcriptome diversity, mechanisms and consequences of APA are vastly understudied compared to mRNA transcription and/or splicing. In coming years, research will likely continue to focus on the three critical areas that were highlighted in the contributions: 1) development of new approaches and technologies to study APA, 2) dissection of the mechanisms that regulate APA and mRNA isoform expression more broadly, and 3) study of the impact of APA on molecular and cellular phenotypes.

While great strides have been made to create novel methodologies for identifying and characterizing APA, many challenges still remain. Terminal ends of mRNA molecules have long been difficult to identify and characterize with unbiased high-throughput approaches, especially in organisms with less well characterized genomes. Previous approaches have focused on targeting 3’ ends of the transcript (i.e., capturing and/or anchoring on the polyA tail), but these approaches often limit investigation of APA usage within the context of the full mRNA molecule. Future approaches will likely build and expand upon newer long-read sequencing approaches, especially as these technologies are refined and become more accessible to a wider array of researchers.

The ability to measure APA events at higher resolution will aid in mapping mechanisms that regulate APA and, more generally, mRNA isoform expression. Recent studies have increasingly shown links between APA, transcriptional, and splicing regulation. The canonical thinking is that APA is regulated, independently of other mRNA processing mechanisms, by separate cleavage and polyadenylation machinery and factors, but evidence for co-regulation of sites across an mRNA molecule challenges this view. Future studies are needed to untangle direct vs. indirect coordination between these mechanisms and address whether full-length isoforms are simply a sum of individual regulatory decisions at the exon level or purposely expressed through the combined actions of seemingly disparate RNA processing mechanisms.

Finally, APA is already implicated in regulating a diverse array of molecular and cellular phenotypes, including gene expression levels, mRNA stability, subcellular RNA localization, and translational efficiency. However, the lack of a good 3′ UTR “code” defining the full cohort of impactful regulatory elements has limited the ability to define all the processes that may be affected by APA. Since 3′ UTRs are not translated, there is no immediate understanding for how different UTR sequences affect downstream molecular phenotypes. Similarly, it is challenging to study the conservation of APA and 3′ UTR function, since the evolution of both *cis-*elements and *trans*-factors regulate APA site usage. Future work will likely focus on characterizing APA in a wider array of organisms and on performing more fine-tuned genetic dissections of individual or combinations of 3’ UTR regulatory elements.

